# Comprehensive Multi-Omics Analysis Identifies FUT1 as a Prognostic and Therapeutic Biomarker Across Pan-Cancer

**DOI:** 10.7150/ijms.108072

**Published:** 2025-02-18

**Authors:** Yunqing Liu, Guanjun Chen, Xiaotian Yuan, Yaxin Chen, YuJie Cui, Ke Cao

**Affiliations:** 1 Department of Oncology, Third Xiangya Hospital of Central South University, Changsha, 410013, China.; 2 Department of Otolaryngology Head and Neck Surgery, Xiangya Hospital of Central South University, Changsha, 410008, China.

**Keywords:** Pan-cancer, FUT1, Tumor microenvironment, Prognostic biomarker Drug sensitivity

## Abstract

Fucosyltransferase 1 encodes a Golgi membrane protein involved in H-antigen precursor production and plays a critical role in tumor-associated glycosylation and angiogenesis. While FUT1 is known to enhance tumor stemness, adhesion, migration, and drug resistance in specific cancers, its role across diverse cancer types and its association with clinical prognosis and molecular features remain unclear. In this study, FUT1 expression was systematically analyzed across 33 cancer types using data from multiple public databases, including CCLE, TCGA, and GTEx. FUT1 expression was found to vary across cancers, correlating with poor prognosis in ACC, BLCA, and COAD and demonstrating high diagnostic accuracy in READ and COAD. Genomic analyses revealed frequent FUT1 amplifications and associations with genomic instability, while functional analyses linked FUT1 to proliferation, metastasis, and EMT pathways. FUT1 expression was also associated with immune microenvironment features, such as immune cell infiltration and stromal scores, and correlated with TMB and MSI. Drug sensitivity analysis indicated that FUT1 expression was linked to lower sensitivity to most drugs but increased sensitivity to tyrosine kinase inhibitors. Experimental validation confirmed that FUT1 knockdown inhibited proliferation, invasion, and migration in bladder, breast, and colorectal cancer cell lines, suggesting a potential role in cancer progression, though further evidence is required to fully establish its oncogenic involvement. These findings highlight FUT1 as a potential prognostic biomarker and provide insights into its biological functions and relevance for developing targeted therapeutic strategies across cancers.

## Introduction

Cancer remains a leading cause of death worldwide, posing significant challenges to public health and quality of life^1^. Recent advances in immunotherapy, particularly immune checkpoint blockade, have transformed cancer treatment, offering improved survival outcomes for many patients^2^. Meanwhile, the development of multi-omics databases, such as The Cancer Genome Atlas, enables comprehensive pan-cancer analyses, facilitating the discovery of novel therapeutic targets and biomarkers that link molecular mechanisms to cancer progression and patient prognosis.

Fucosyltransferases are key enzymes in glycosylation, a critical post-translational modification involved in cell adhesion, migration, and immune regulatio^3^-^5^. Among the 11 FUT genes identified in humans, Fucosyltransferase 3 and Fucosyltransferase 6 promote cancer cell adhesion and migration via sialyl Lewis antigens and TGF-β-mediated epithelial-mesenchymal transition^6^, while Fucosyltransferase 4 (FUT4) is implicated in immune regulation and poor prognosis in lung adenocarcinoma patients through its influence on PD-1 expression^7^. Fucosyltransferase 1 (FUT1), a crucial member of the fucosyltransferase family, is encoded by the H gene in hematopoietic tissues^8^,^9^. The protein encoded by FUT1 primarily functions in the Golgi apparatus. As a type II transmembrane protein, it facilitates glycosylation reactions both inside and outside the cell, transferring monosaccharides to proteins and lipids^10^. Beyond its role in glycosylation, FUT1 is essential for T cell development and regulating inflammatory responses^11^. During tumorigenesis, FUT1 promotes tumor stemness, adhesion, migration, and drug resistance through the AKT/mTOR/4EBP1 signaling pathway^12^. Moreover, FUT1 is involved in the proliferation of breast cancer cells and the development of paclitaxel resistance in ovarian cancer cells^13^,^14^. These findings underscore the critical role of FUT1 in tumor progression and its potential as a therapeutic target. However, its widespread function and differential roles across various cancers have yet to be fully explored.

Despite these insights, most studies on FUT1 have focused on single cancer types, leaving its broader role across the pan-cancer spectrum and its potential connection to immune-related features underexplored. To address this gap, this study provides a comprehensive analysis of FUT1 expression across 33 cancer types using data from TCGA, GTEx, CCLE, and other multi-omics databases. We examined the associations between FUT1 expression and DNA methylation, immune cell infiltration, tumor mutational burden (TMB), microsatellite instability (MSI), and patient prognosis. Enrichment analyses were conducted to further explore its biological functions, and experimental validation was performed in three cancer cell lines.

Our findings demonstrate that FUT1 serves as a predictive biomarker in multiple malignancies, strongly correlating with tumor progression and patient prognosis. While FUT1 also exhibits associations with immune-related features such as TMB, MSI, and tumor-infiltrating immune cells, these results suggest its role in immune modulation may be secondary to its primary functions in tumor progression. This study highlights FUT1 as a promising pan-cancer therapeutic target and prognostic biomarker, offering valuable insights into cancer treatment strategies.

## Methods and Materials

### Pan-cancer data collection and processing

The expression of FUT1 across different cancer cell lines was analyzed using the Cancer Cell Line Encyclopedia (CCLE, https://sites.broadinstitute.org/ccle)^15^. Pan-cancer mRNA expression profiles, clinical information, survival data, somatic mutation data, CNV data, and DNA methylation data (HumanMethylation450K) were obtained from the UCSC Xena platform (http://xena.ucsc.edu/) using data from The Cancer Genome Atlas (TCGA, https://www.cancer.gov/tcga) and Genotype-Tissue Expression (GTEx, https://commonfund.nih.gov/GTEx) database. Single-cell expression profiles of FUT1 in various cell types were analyzed using the TISCH database (https://tisch.comp-genomics.org)^16^,^17^. Transcriptome data were normalized using the log2(TPM + 1) transformation method. Detailed information on the nomenclature and abbreviations of the 33 cancer types included in this study is provided in Table S1.

### Survival analysis and diagnostic model

The relationship between FUT1 mRNA expression levels and prognosis was assessed for overall survival (OS), disease-specific survival (DSS), disease-free interval (DFI), and progression-free interval (PFI). Kaplan-Meier analysis was performed using the 'survival' R package, with optimal cutoff values determined by 'survminer'. To prevent bias, the ratio of high- to low-expression groups was set to ≥0.2, and significance was evaluated using log-rank tests. Cox proportional hazards modeling was used to calculate hazard ratios (HR) and 95% confidence intervals (CI) for each cancer type. Results were visualized with 'ComplexHeatmap'.Diagnostic performance was evaluated with ROC analysis using the 'pROC' R package, including AUC calculations, 95% CIs, and smoothed ROC curves.

### Genomic alterations and genomic instability analysis

The mutation frequency of FUT1 across cancers was analyzed using the cBioPortal database (https://www.cbioportal.org/), with a differential mutation frequency plot generated. The Cancer Type Summary module in cBioPortal was employed to evaluate the frequencies of three genomic alteration types—mutations, amplifications, and deep deletions—across multiple cancer type^18^-^20^. The Kruskal-Wallis rank-sum test was used to assess statistical differences in FUT1 expression among different CNV groups across cancers. Somatic mutation data from the TCGA database were analyzed using the 'maftools' R package.

The correlation between FUT1 expression and genomic instability indicators—including Aneuploidy, Homologous Recombination Defects, Tumor Ploidy, SNV Neoantigens, Nonsilent Mutation Rate, and Silent Mutation Rate—was calculated using the cor.test function^21^. Correlation coefficients were visualized using radar charts generated with the 'fmsb' R package.

### Correlation analysis between mRNA expression levels, DNA methylation, and RNA modification patterns

Pan-cancer DNA methylation data were obtained, and probe annotations were performed using the 'ChAMP' R package. Spearman correlations between FUT1 expression and DNA methylation beta values at various genomic loci were calculated using the cor.test function. RNA modification patterns, along with associated genes and functional annotations, were retrieved from the RM2Target database (http://rm2target.canceromics.org/#/home), which is a comprehensive resource for targets of RNA modification writers, erasers, and readers (WERs)^22^. The expression levels of these genes were extracted and analyzed to assess their correlation with FUT1 expression.

### mRNA expression analysis in carcinogenic pathways

The CancerSEA database (http://biocc.hrbmu.edu.cn/CancerSEA/home.jsp) was used to obtain 14 functional state-related gene sets across different cancer types. Hallmark gene sets (h.all.v7.2.symbols.gmt) were retrieved from the Msigdb database (http://software.broadinstitute.org/gsea/msigdb). GSVA analysis was performed using the 'GSVA' package in R, and enrichment scores for each gene set in each sample were calculated to create an enrichment score matrix. The correlation between FUT1 mRNA expression levels and GSVA scores of various carcinogenic pathways was then assessed.

### Protein-protein interaction network construction

The FUT1 protein interaction network was constructed using STRING (https://string-preview.org/) and GeneMANIA (http://genemania.org/)^23^,^24^. Cytoscape was used for weight calculation and network visualization. Furthermore, the 'gprofile2' R package was applied to perform GO and KEGG functional enrichment analysis of the interacting proteins.

### Immune microenvironment correlation analysis

The 'TCGAplot' R package was used to analyze the correlation between FUT1 mRNA expression levels and immune-related genes across various cancers, including immune checkpoint genes, chemokines, chemokine receptors, immune stimulators, and immune inhibitors. The correlation between mRNA expression levels and immune infiltration was also assessed, including immune cell proportions and immune scores. Additionally, the relationship between mRNA expression levels and TMB and MSI was explored.

### Drug sensitivity prediction

The TIDE database (http://tide.dfci.harvard.edu/download/) was used to obtain immunotherapy trial studies^25^,^26^. FUT1 expression levels were compared between responders (R) and non-responders (NR) to evaluate its predictive value for immunotherapy response. The statistical significance of differences was calculated using the Wilcoxon rank-sum test, implemented in R with the wilcox.test function.

For pan-cancer analysis, the Connectivity Map (CMap) database (https://portals.broadinstitute.org/cmap/) was utilized. The XSum (eXtreme Sum) optimal feature-matching method was applied to compare gene-related features with CMap gene signatures, generating similarity scores for 1,288 compounds. These scores were used to evaluate the potential functional effects of compounds on FUT1.

### Cell culture and transfection

UMUC3, MDA-MB-361 and HCT116 were purchased from the Chinese Academy of Sciences Committee Type Culture Collection Cell Bank (Shanghai, China). Cells were grown in DMEM (Pricella, cat#PM150210) and Leibovitz's L-15 (Pricella, cat#PM151010), 10% certified heat-inactivated FBS (Pricella, cat#164210-50), 100 U/mL penicillin-streptomycin, and 5% carbon dioxide in an incubator at 37°C. GenePharma obtained siRNA and negative controls (Suzhou, China). FUT1 was targeted by siRNA with the sequence 5′-GUGGGCAUUAAUGCAGACUTT-3′, while siRNA-control had the sequence 5′-UUCUCCGAACGUGUCACGUTT-3′. The siRNA-FUT1 and siRNA control gene were cotransfected with the above three cells using liposome 2000 (Invitgen, cat#11668030).

### Quantitative real-time PCR (qRT-PCR)

After total RNA was extracted from cells using the Steadypure Quick RNA Extraction Kit (Accurate Biology, cat#AG21023), RNA purity was assessed spectrophotometrically (A260/A280 > 1.8) to ensure RNA quality for subsequent experiments. Subsequently, 1 μg of total RNA was reverse-transcribed into cDNA using the HiScript II Q RT SuperMix (Vazyme, cat# R223). qRT-PCR was performed with the SYBR Green Master Mix (Vazyme, cat# Q711). Relative RNA expression levels were calculated using the 2^-△△Ct^ method, normalized to GAPDH as the internal control. The primer sequences used in this study were as follows: FUT1 (forward: 5'-CTTCCTGCTAGTCTGTGTCCT-3', reverse: 5'-ATTGGGGTAGACAGTCCAGGT-3') and GAPDH (forward: 5'-GTCAGCCGCATCTTCTTT-3', reverse: 5'-CGCCCAATACGACCAAAT-3').

### CCK-8 assay

UMUC3, MDA-MB-361, and HCT116 cells (2,500 cells/well) were seeded in 96-well plates in 100 μL of medium and cultured for the designated time periods. After adding 10 μL of the CCK-8 reagent (Biosharp Life Sciences) to each well, the plates were incubated for 2 hours. The optical density (OD) was measured at 450 nm using a microplate reader.

### Colony formation assay

UMUC3, MDA-MB-361, and HCT116 cells were seeded at a density of 1,000 cells per well in 6-well plates and incubated for 2-3 weeks to allow colony formation. The colonies were then fixed with paraformaldehyde, stained with crystal violet, and imaged. Colonies containing at least 50 cells were counted under a microscope.

### Transwell matrigel invasion assay

The Transwell chambers were pre-coated with Matrigel to assess cell invasion. Matrigel was diluted with serum-free cell culture medium, mixed thoroughly, and 50 μL of the mixture was added to each chamber insert. The inserts were incubated in a CO₂ incubator for 2 hours, followed by the addition of 100 μL serum-free medium to each insert, which was then placed in the incubator for 30 minutes. After 24 hours of siRNA transfection, the three cell lines were washed with PBS and serum-free DMEM. The lower chamber was filled with 600 μL DMEM containing 20% FBS as a chemoattractant, and 200 μL of serum-free cell suspension was added to the upper chamber. The cells were incubated for 24 hours in a CO₂ incubator, after which the non-invading cells on the upper surface of the insert membrane were removed. The cells that had invaded to the lower surface of the Transwell membrane were fixed, stained with 0.1% crystal violet, and washed. The stained cells were counted under a microscope from six randomly selected fields to determine the number of positively stained invasive cells.

### Wound-healing assay

UMUC3, MDA-MB-361, and HCT116 cells were cultured in 6-well plates until they formed a 100% confluent monolayer. A sterile 10 μL pipette tip was used to create a straight scratch in the cell monolayer. The medium was then replaced with serum-free medium after washing to remove debris. Wound closure was monitored by capturing images at 0 and 48 hours.

### Statistical analysis

R (v4.2.2) and GraphPad Prism (v.9.2) were utilized for statistical data calculations. The Student's t-test was utilized for difference analysis when comparing the two groups in the experimental setup. In the CCK8 experiment, a two-way ANOVA was used to compare differences. For all statistical studies, a two-tailed *p* < 0.05 was considered statistically (*p<0.05, **p < 0.01, ***p < 0.001, and **** p < 0.0001).

## Results

### Pan-cancer analysis of FUT1 expression in cells and tissues

**Figure 1:**
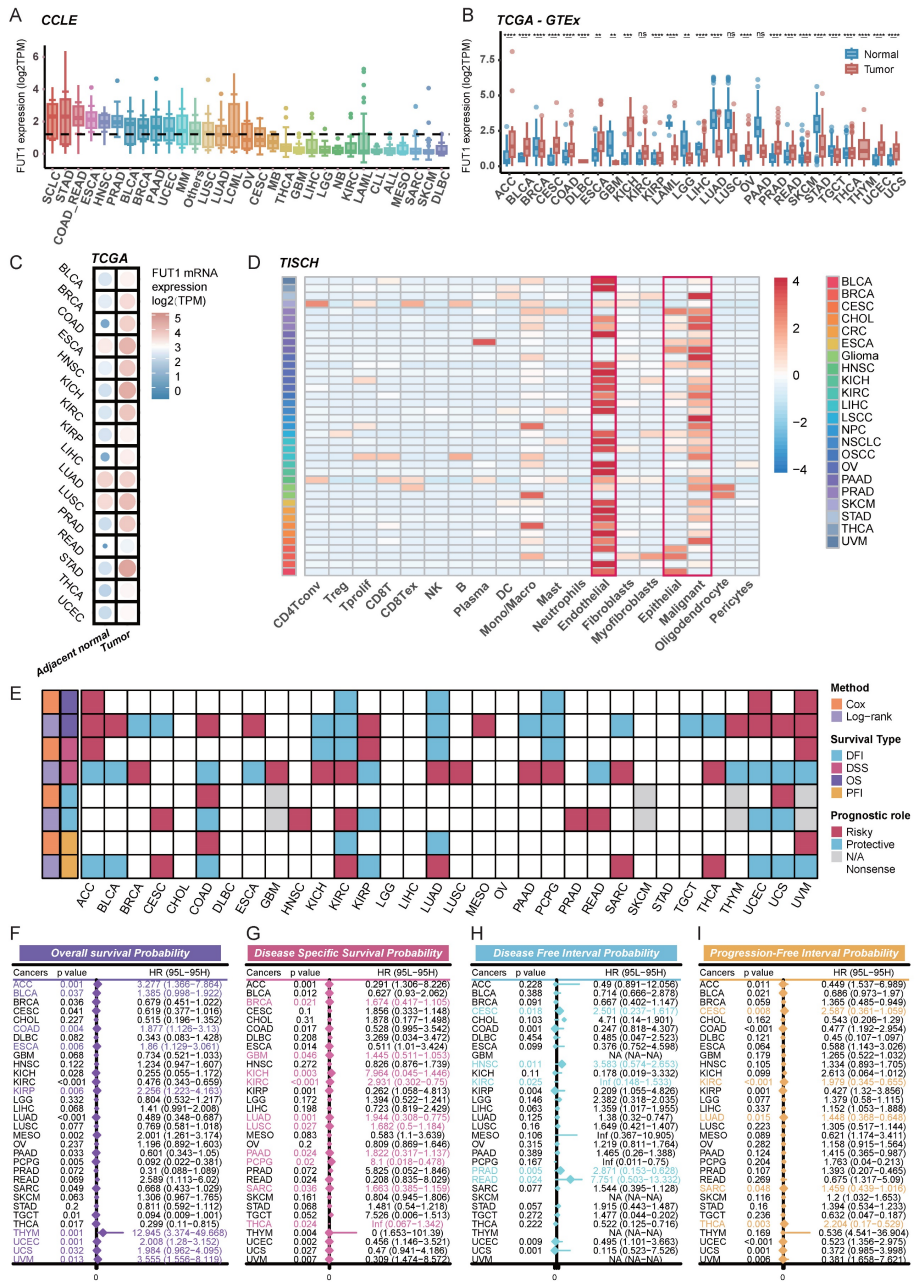
** FUT1 expression patterns and prognostic significance across pan-cancer.** (A) FUT1 mRNA expression levels in pan-cancer cell lines based on the CCLE database. (B) Boxplot comparing FUT1 mRNA expression levels between tumor and normal tissues across various cancers. Data were integrated from TCGA and GTEx databases. Statistical significance was assessed using Wilcoxon rank-sum test (*p < 0.05, **p < 0.01, ***p < 0.001, and ****p < 0.0001). (C) Bubble plot illustrating differential FUT1 mRNA expression levels between tumor and adjacent normal tissues in TCGA pan-cancer data. (D) Heatmap depicting single-cell level expression of FUT1 across different cancers using the TISCH database. Data were clustered by cell type and tissue of origin. (E) Heatmap summarizing the prognostic role of FUT1 in pan-cancer using Cox regression and log-rank tests. Survival types analyzed include OS, DSS, DFI, and PFI. Red and blue indicate risky and protective roles, respectively. (F-I) Forest plots displaying the hazard ratios (HR) with 95% confidence intervals for FUT1 in (F) overall survival (OS), (G) disease-specific survival (DSS), (H) disease-free interval (DFI), and (I) progression-free interval (PFI) across cancers. HRs were derived from univariate Cox regression analysis, with significant results annotated.

RNA expression profiles of 1,019 cancer cell lines were retrieved from the CCLE database to analyze FUT1 expression across cancers. The highest levels of FUT1 expression were observed in SCLC, while certain gastrointestinal cancers, including STAD, COAD_READ, and ESCA, also exhibited relatively high expression (Figure 1A).

Next, we analyzed FUT1 mRNA expression across various human cancer types using TCGA data and compared it to corresponding normal tissues from the GTEx database. The results showed that FUT1 mRNA expression was significantly higher in many cancers compared to their normal counterparts. Notably, KICH exhibited the highest FUT1 expression, with a median expression of 6.09 TPM, representing a 6.16-fold increase compared to benign kidney tissue. In contrast, in lung cancers, including LUAD and LUSC, FUT1 expression was significantly higher in normal tissues than in tumor tissues (Figure 1B). In COAD, the FUT1 mRNA expression level increases with advancing pathological stage, whereas in KIRC, the expression follows an opposite trend. Additionally, FUT1 expression is found to be associated with age in certain tumors (Figure S1).

Further analysis of paired tumor and adjacent normal tissues from the TCGA database revealed that FUT1 mRNA expression was generally higher in tumor tissues across most cancer types compared to adjacent normal tissues (Figure 1C).

To investigate FUT1 mRNA expression at the single-cell level, we analyzed pan-cancer single-cell RNA-seq data from TISCH. The results indicated that FUT1 was predominantly expressed in endothelial cells across most cancers. Additionally, it was also expressed in epithelial cells, with malignant epithelial cells exhibiting significantly higher expression than normal epithelial cells. In some cancers, FUT1 was also expressed in immune cell populations, with monocyte and macrophage subsets showing higher expression compared to other immune cell types (Figure 1D).

### FUT1 as a diagnostic and prognostic tool for different cancers

To explore the association between FUT1 mRNA expression levels and survival prognosis, we performed survival analysis using Kaplan-Meier (Logrank Mantel-Cox test) and Cox regression. Analysis of OS, DSS, PFI, and DFI across pan-cancer types revealed that high FUT1 expression is a risk factor for poor survival prognosis in certain cancers (Figure 1E). Notably, high FUT1 expression was associated with poorer OS in ACC (HR = 3.277, logrank-test *p* = 0.001), BLCA, and COAD. Additionally, higher FUT1 expression was linked to poorer DSS in BRCA (HR = 1.674, logrank-test *p* = 0.021) and GBM (Figure 1F-I). Kaplan-Meier survival curves showed that higher FUT1 expression correlated with lower OS probability in nine cancer types (Figure S2).

Further evaluation of the diagnostic value of FUT1 in pan-cancer revealed that in diagnostic ROC models for READ and COAD, the area under the curve (AUC) reached 0.999 and 0.985, respectively. The ROC curves indicated that FUT1 can serve as a diagnostic biomarker for certain cancers (Figure S3).

### Genomic alterations and genomic instability analysis of FUT1

**Figure 2:**
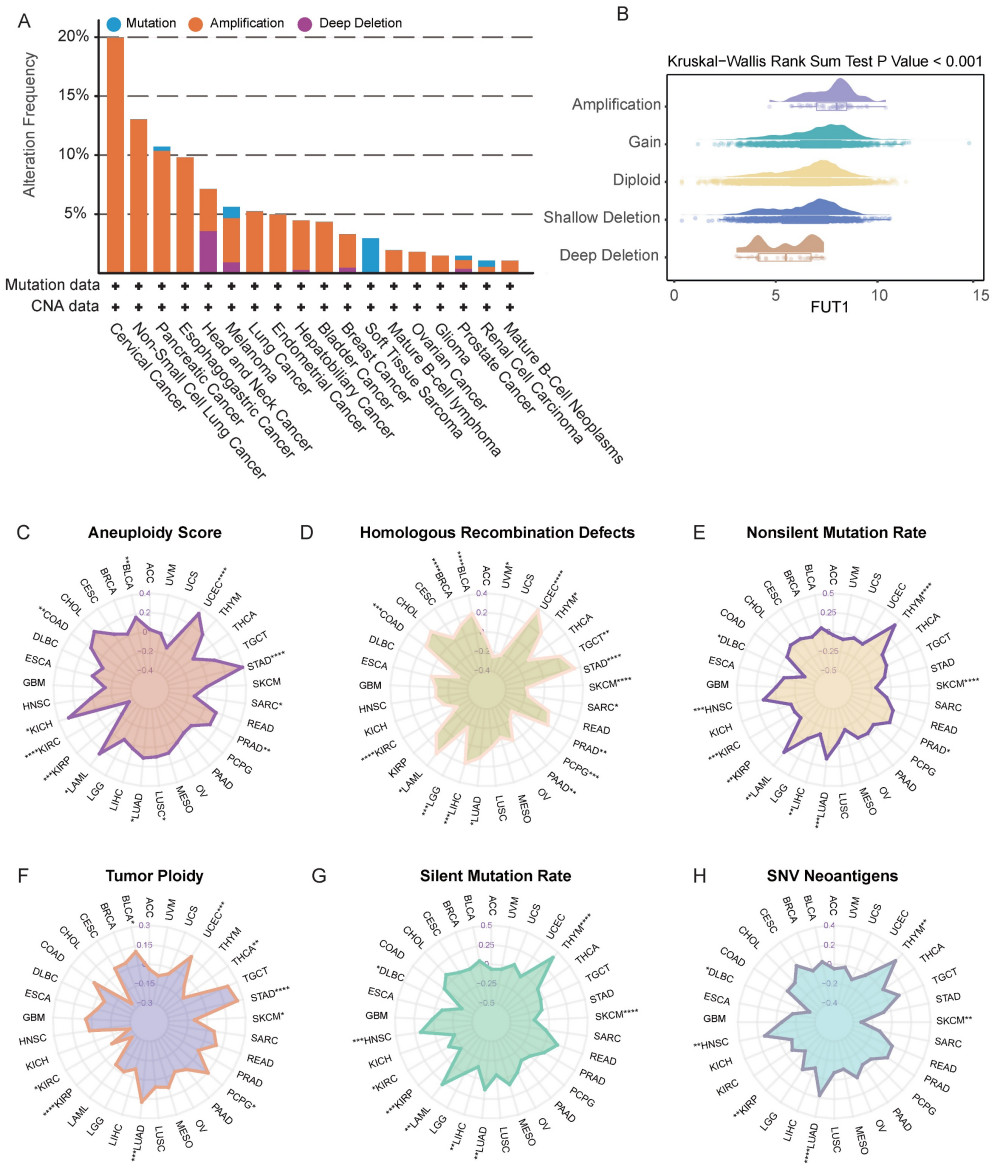
** The association of FUT1 expression with genomic instability across pan-Cancer.** (A) Pan-cancer analysis of genomic alterations in FUT1, including mutations, amplifications, and deep deletions, using TCGA data. (B) Differential FUT1 expression across various CNV (Copy Number Variation) types. The box represents the interquartile range (IQR), the line inside the box indicates the median, and whiskers represent the range. Statistical differences were assessed using the Kruskal-Wallis rank-sum test. (C-H) Radar plots displaying Spearman correlation coefficients between FUT1 expression and genomic instability scores: Aneuploidy Score (C), Homologous Recombination Defects (D), Silent Mutation Rate (E), Non-Silent Mutation Rate (F), SNV Neoantigens (G), and Tumor Ploidy (H). Each axis represents the magnitude of correlation, and significance levels are indicated as ****p < 0.0001, ***p < 0.001, **p < 0.01, and *p < 0.05.

Genomic strategies provide powerful tools for cancer analysis^16^. To assess potential genomic alterations of FUT1 in specific cancers, we performed a pan-cancer analysis of FUT1 copy number variations (CNVs) and genomic instability-related scores. FUT1 amplification was the most common genomic alteration, with the highest alteration frequency observed in cervical cancer. Deep deletions were primarily found in head and neck cancer and melanoma, while mutations were most common in soft tissue sarcoma (Figure 2A-B).

Further genomic exploration of the FUT gene family revealed that the overall mutation frequency of FUT genes was higher in COAD, SKCM), and UCEC (Figure S4A). FUT9 exhibited a high frequency of genomic alterations across pan-cancer types (Figure S3B). To further investigate the genetic aberrations of FUT genes in cancer, we analyzed the percentage of somatic copy number alterations (SCNAs). The results showed that SCNAs occurred at a high frequency in most cancer types (Figure S4C). Notably, somatic copy number alterations of FUT10 were highly correlated with FUT1 mRNA expression in most cancers (Figure S3D).

Correlation analysis between FUT1 mRNA expression levels and various genomic instability scores, including aneuploidy, homologous recombination defects, tumor ploidy, SNV neoantigens, nonsilent mutation rate, and silent mutation rate, revealed a positive correlation in some cancers. This suggests that higher FUT1 expression is associated with increased chromosomal instability in these patients (Figure 2C-H).

### Correlation analysis of FUT1 expression with DNA methylation and RNA modification patterns

**Figure 3:**
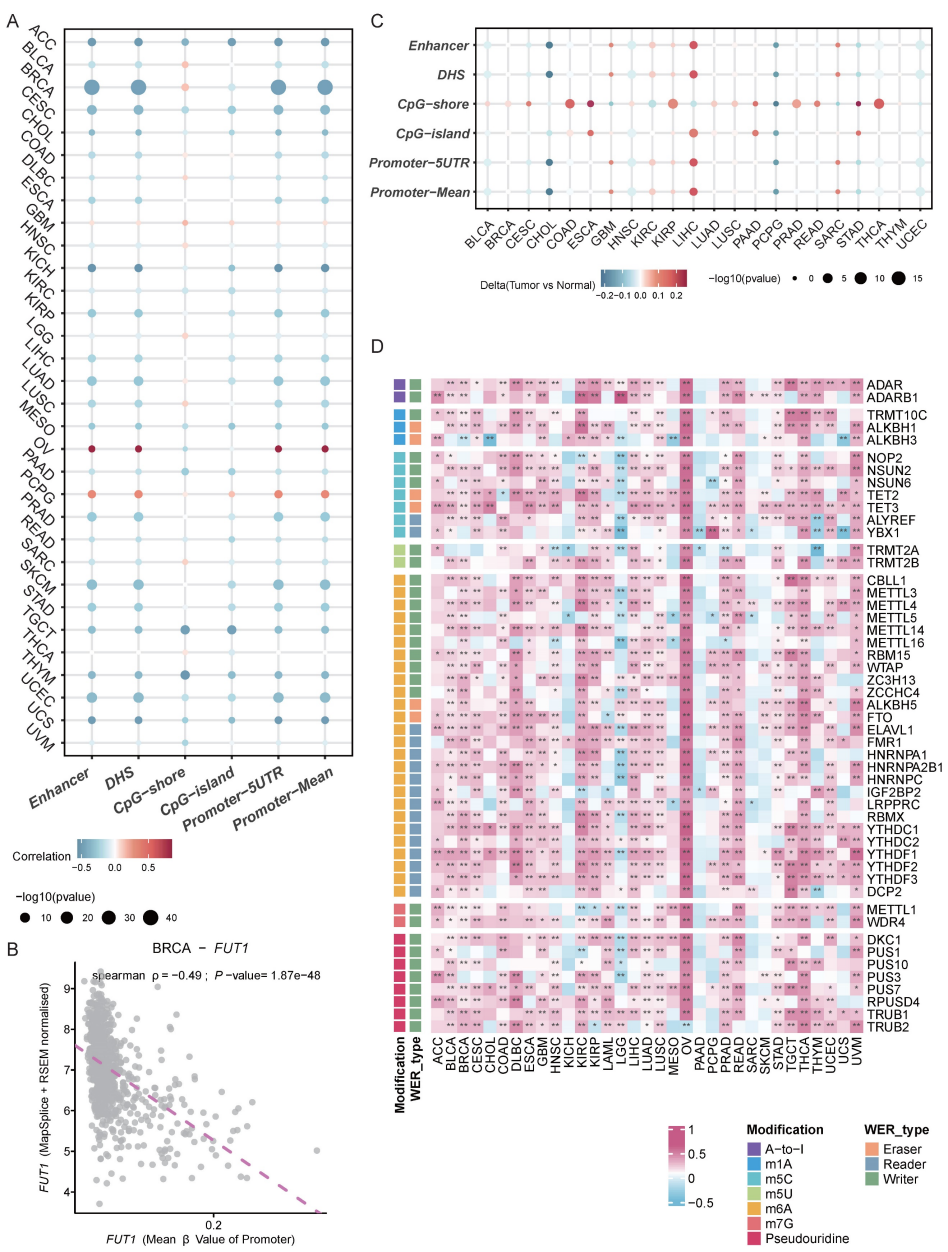
** Association between FUT1 DNA methylation, RNA modification patterns, and mRNA expression.** (A) Heatmap showing the correlation between FUT1 DNA methylation levels and mRNA expression across cancers. (B) Scatter plot illustrating the relationship between FUT1 DNA methylation levels and mRNA expression in the TCGA-BRCA cohort. Spearman correlation coefficient (ρ) and statistical significance are annotated. (C) Heatmap displaying differential DNA methylation levels of FUT1 between tumor and adjacent normal tissues across cancers. Genes with hypermethylation and hypomethylation are marked in red and blue, respectively (Wilcoxon rank-sum test). (D) Heatmap summarizing the correlation between FUT1 mRNA expression and the expression of key genes involved in seven RNA modification pathways (A-to-I, m1A, m5C, m5U, m6A, m7G, and pseudouridine). Statistical significance is denoted as ****p < 0.0001, ***p < 0.001, **p < 0.01, and *p < 0.05.

To explore the potential reasons for the differential FUT1 mRNA expression levels between tumor and adjacent normal tissues in various cancers, we further investigated the impact of DNA methylation and RNA modification patterns on FUT1 expression. Annotation of FUT1 methylation probes revealed that in most cancers, the methylation levels of DNA promoters and enhancers were negatively correlated with mRNA levels, such as in BRCA (ρ=-0.49, *p* =1.87e-48). However, in ovarian cancer (OV) (ρ=0.85, *p*=0.00342) and PCPG (ρ=0.35, *p*=1.25e-06), DNA promoter methylation levels were positively correlated with FUT1 mRNA expression (Figure 3A-B, Figure S4).

We also examined the differences in methylation levels between tumor and adjacent normal tissues at various loci across different cancers. The results showed that in 22 cancer types, FUT1 DNA methylation patterns were complex. Notably, the methylation levels of CpG-shores were higher in tumor tissues, with the overall methylation level of FUT1 in LIHC tumor tissues being higher than in adjacent normal tissues (Figure 3C).

Unlike DNA and histone modifications, which regulate gene expression through reversible epigenetic modifications, RNA methylation represents another layer of gene expression regulatio^27^. We analyzed the correlation between key genes in seven RNA modification patterns and FUT1 mRNA expression levels. The results indicated a positive correlation between the expression levels of key RNA modification genes and FUT1 expression in most cancers, with a particularly strong positive correlation observed in OV (Figure 3D, Figure S4).

### FUT1 involvement in multiple carcinogenic pathways

**Figure 4:**
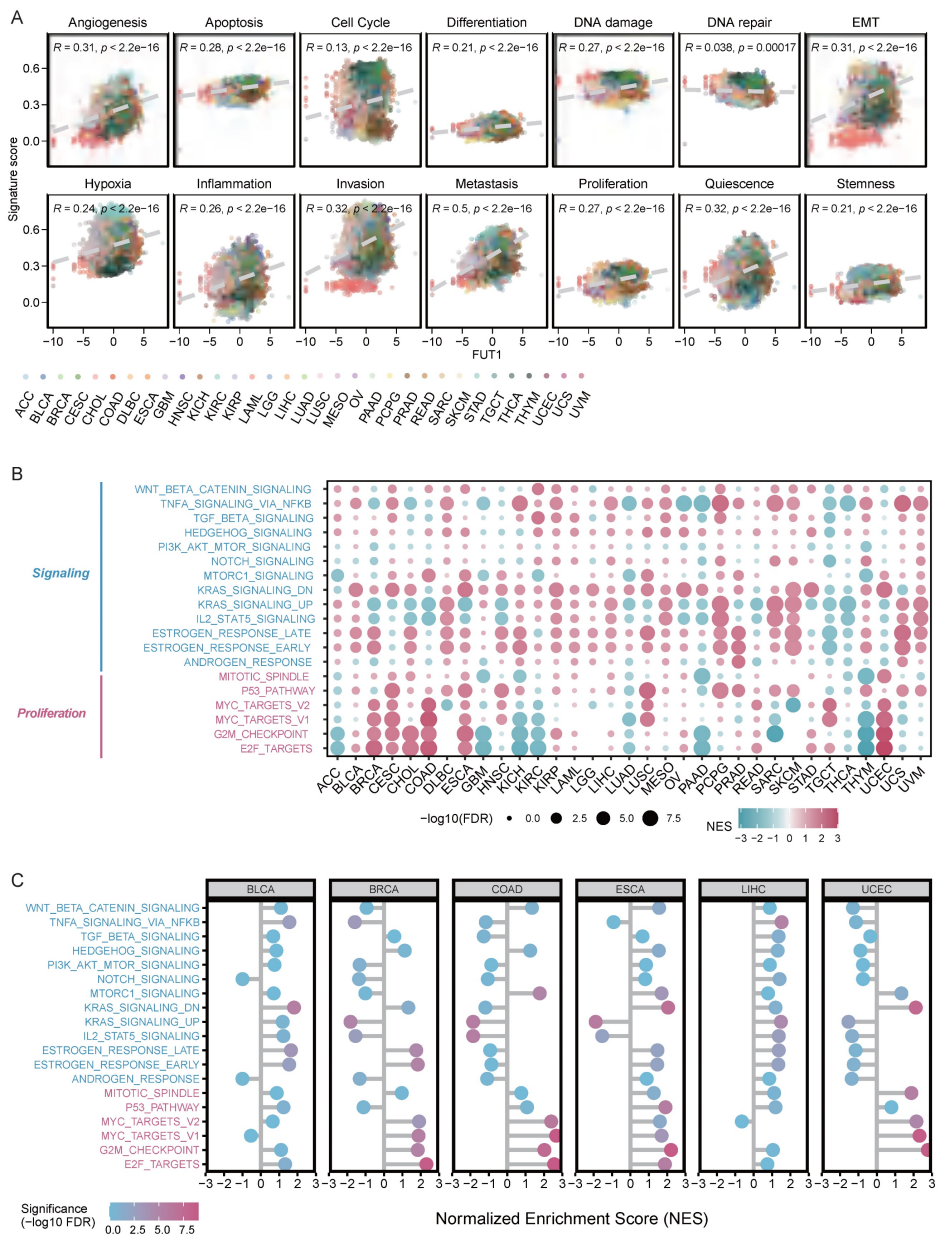
** Exploring the functional role of FUT1 in pan-cancer tumorigenesis.** (A) Correlation between FUT1 expression and 14 tumor progression signature scores across cancers. Spearman correlation coefficients (R) and p-values are shown. (B) Enrichment analysis of signaling and proliferation pathways in tumors with high and low FUT1 expression. Pathway significance is evaluated using normalized enrichment scores (NES) from GSEA. (C) Correlation analysis between FUT1 expression and NES values for signaling and proliferation pathways in BLCA, BRCA, COAD, ESCA, LIHC, and UCEC tumors.

The CancerSEA database categorizes 14 different functional states of tumor cell^28^. We analyzed the correlation between FUT1 expression levels and the functional state scores of these 14 tumor cell pathways. Our results revealed a strong positive correlation between high FUT1 expression and metastasis scores across pan-cancer types (R=0.5, *p* < 2.2e-16), as well as positive correlations with scores for other pathways, including quiescence, invasion, and epithelial-mesenchymal transition (EMT) (Figure 4A).

Enrichment analysis of the Hallmark gene sets showed that in BLCA, BRCA, cervical CESC, CHOL, COAD, and UCEC, high FUT1 expression was positively correlated with multiple pathways in the proliferation gene set. Moreover, high FUT1 expression had a significant impact on estrogen response, KRAS signaling, and TNF-α signaling via NF-κB pathways in most cancer types (Figure 4B).

Further analysis of the correlation between FUT1 expression levels and normalized enrichment scores (NES) for multiple proliferation and signaling pathways in BLCA, BRCA, COAD, ESCA, LIHC, and UCEC showed that in BRCA, COAD, and ESCA, high FUT1 expression was strongly positively correlated with the overall enrichment of proliferation pathways (Figure 4C).

### Construction of FUT1 protein interaction and co-expression network

**Figure 5:**
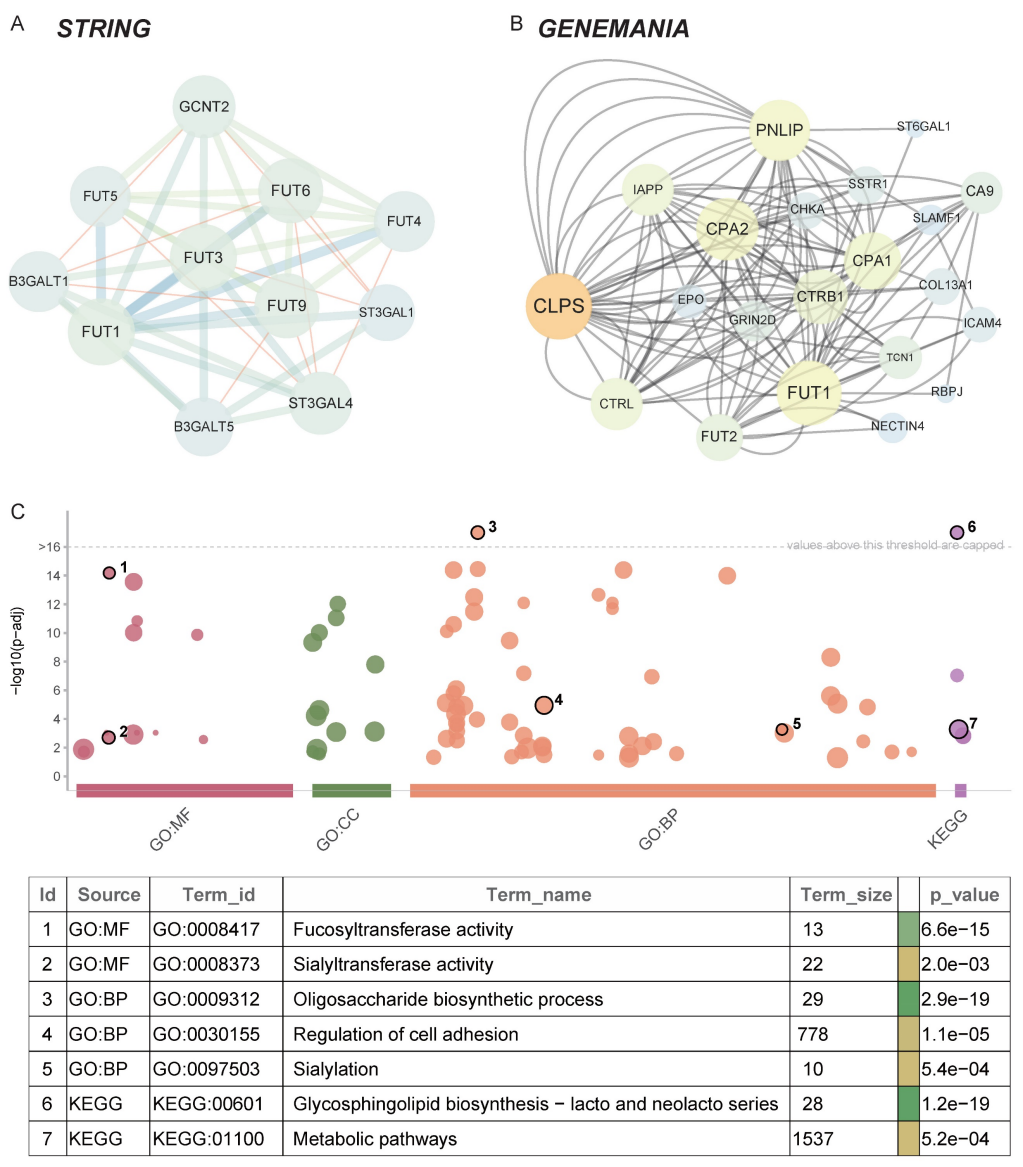
** Protein-protein interaction network and functional enrichment analysis of FUT1.** (A-B) Protein-protein interaction (PPI) networks of FUT1 visualized using (A) the STRING database and (B) the GeneMANIA platform, showing interacting proteins and associated pathways. (C) Gene Ontology (GO) and KEGG enrichment analysis of FUT1-interacting proteins.

The construction of a protein-protein interaction (PPI) network provides further insights into the biological functions of FUT1 in pan-cancer. Using the STRING and GeneMANIA databases, we identified 31 interacting proteins, including FUT1 (Figure 5A-B).

GO and KEGG enrichment analyses revealed that FUT1 and its interacting proteins are primarily involved in metabolism-related pathways, such as fucosyltransferase activity, sialylation, and glycosphingolipid biosynthesis. Additionally, these proteins are associated with pathways regulating cell adhesion, which aligns with the strong positive correlation observed between FUT1 expression and invasion and EMT pathway scores described earlier (Figure 5C).

### Correlation of FUT1 with the tumor microenvironment

**Figure 6:**
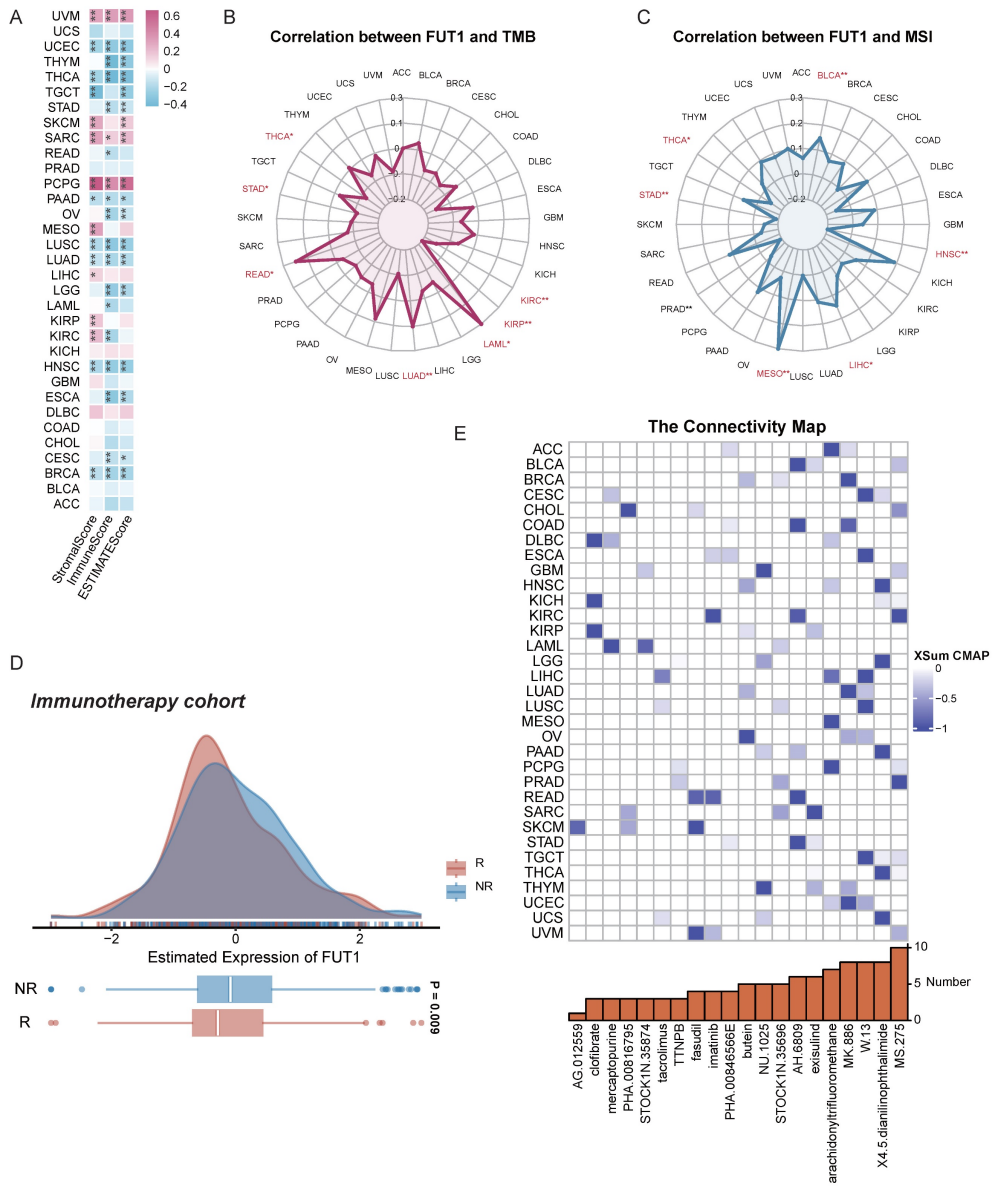
** FUT1 as a pan-cancer biomarker for predicting therapeutic sensitivity.** (A) Correlation between FUT1 expression and ImmuneScore, StromalScore, and ESTIMATEScore across various cancers. (B-C) Radar charts representing pan-cancer analyses of the correlation between FUT1 expression and (B) tumor mutational burden (TMB) and (C) microsatellite instability (MSI). (D) Differential expression of FUT1 in non-responders (NR) and responders (R) in immunotherapy cohorts. (E) Identification of potential small-molecule drugs to reverse FUT1 dysregulation using the Connectivity Map (CMap) database.

To explore whether FUT1 expression influences the tumor microenvironment (TME), we analyzed its association with stromal and immune cell components. The results indicated that FUT1 expression was negatively correlated with StromalScore and ImmuneScore in most cancers, but showed strong positive correlations in UVM, SKCM, SARC, and PCPG (Figure 6A).

Further analysis of immune cell infiltration in the TME revealed that FUT1 expression was generally negatively correlated with T cell infiltration across most cancers, although the differences were not statistically significant (Figure S6A). Correlation analysis of FUT1 expression with immune-related functional gene sets, including immune checkpoint genes, immunostimulators, immunoinhibitors, chemokines, and chemokine receptors, showed significant positive correlations in cancers such as UVM, PCPG, KIRP, and LIHC. In contrast, negative correlations were frequently observed in BRCA, LUSC, and head and HNSC (Figures S6B and S7A-D).

### FUT1 and drug sensitivity analysis

Given the abundance of genetic mutations in tumors and their potential impact on prognosis and therapeutic outcomes, we evaluated the correlation between FUT1 expression and TMB as well as MSI. A strong positive correlation between FUT1 and TMB was detected in READ (Figure 6B), while a similar correlation with MSI was observed in MESO (Figure 6C).

To assess the association between FUT1 expression and responses to immune checkpoint inhibitors (ICIs), analysis of immunotherapy cohorts revealed higher FUT1 expression in NR patients (Figure 6D, Figure S8, Table S2). Additionally, correlations between gene expression and the half-maximal inhibitory concentration (IC50) of measured antagonists in the GDSC1 and GDSC2 databases indicated that FUT1 expression was positively correlated with IC50 for most drugs, suggesting that higher FUT1 expression is associated with lower drug sensitivity in cell lines. However, for small molecule tyrosine kinase inhibitors (TKIs), including Ibrutinib, Afatinib, Osimertinib, Gefitinib, and Sapitinib, higher FUT1 expression correlated with increased drug sensitivity (Figure S9).

Further predictions using the CMap identified potential drugs targeting FUT1. In certain cancers, MS.275 was suggested to reverse molecular characteristics caused by dysregulated FUT1 expression, potentially mitigating its pro-tumorigenic effects (Figure 6E).

### Preliminary validation of FUT1 in bladder, breast and colon cancer cells

**Figure 7:**
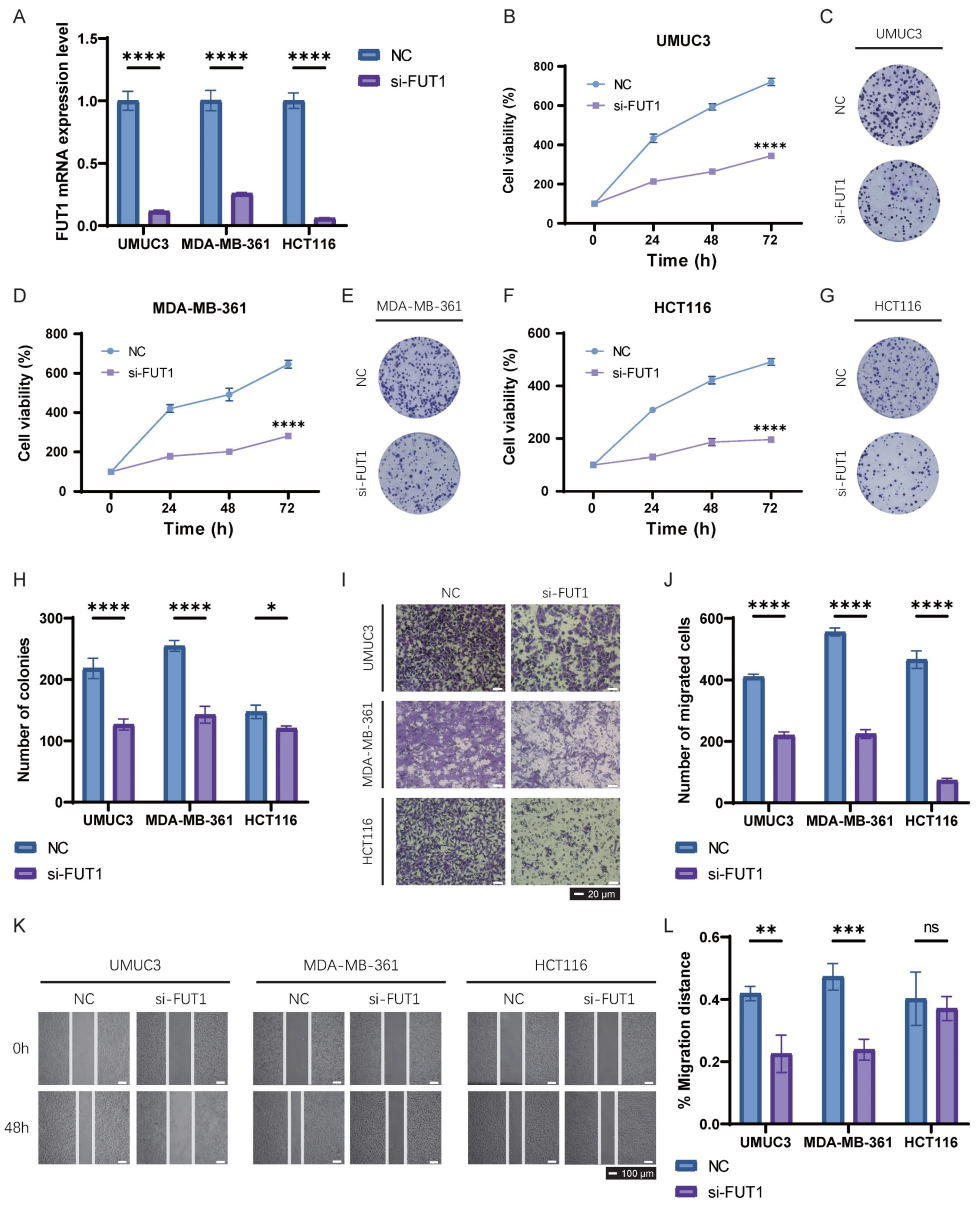
** Functional validation of FUT1 in UMUC3, MDA-MB-361, and HCT116 cell lines.** (A) Validation of siRNA-mediated FUT1 knockdown efficiency using qRT-PCR. (B-C) Effect of FUT1 knockdown on UMUC3 cell proliferation, as assessed by (B) CCK-8 assay and (C) colony formation assay. (D-E) Effect of FUT1 knockdown on MDA-MB-361 cell proliferation, as assessed by (D) CCK-8 assay and (E) colony formation assay. (F-G) Effect of FUT1 knockdown on HCT116 cell proliferation, as assessed by (F) CCK-8 assay and (G) colony formation assay. (H) Bar charts showing the statistical differences in colony formation between NC (negative control) and si-FUT1 groups in UMUC3, MDA-MB-361, and HCT116 cells. (I-J) Invasion assays comparing NC and si-FUT1 groups in the three cell lines. (K-L) Wound healing assays comparing migration ability between NC and si-FUT1 groups in the three cell lines.

We selected three cancer cell lines from distinct cancer types—UMUC3, MDA-MB-361 and HCT116—to perform basic experiments validating the specific biological functions of FUT1 in BLCA, BRCA, and COAD. FUT1 was transiently silenced in these cell lines using siRNA, and qRT-PCR was employed to confirm knockdown efficiency. FUT1 expression was significantly reduced in all three cell lines following siRNA transfection (Figure 7A).

CCK-8 and colony formation assays demonstrated that FUT1 knockdown significantly inhibited proliferation in UMUC3 and MDA-MB-361 cells, while the inhibitory effect on HCT116 cell proliferation was weaker (Figure 7B-H). Transwell invasion assays showed that FUT1 knockdown markedly reduced the invasive capacity of UMUC3, MDA-MB-361, and HCT116 cells, with a particularly pronounced difference observed in HCT116 cells (Figure 7I-J).

Wound healing assays revealed that FUT1 knockdown impaired the migratory ability of UMUC3 and MDA-MB-361 cells, whereas the migration capacity of HCT116 cells following injury was not significantly affected (Figure 7K-L).

## Discussion

FUT1, a rate-limiting enzyme in Lewis y antigen synthesis, plays a crucial role in tumor progression. Its suppression reduces Lewis y levels and inhibits cancer growth^29^. This study represents the first comprehensive pan-cancer analysis of FUT1, revealing its differential expression across tumor types and its prognostic significance. FUT1 was upregulated in 14 cancers and downregulated in 11, consistent with findings in liver, kidney, ovarian, colorectal, breast, prostate, and lung cancers^12^,^13^,^30^-^34^. Kaplan-Meier survival analysis showed that FUT1 overexpression correlates with poor prognosis in BRCA and COAD, aligning with prior studies^12^.

Conversely, FUT1 downregulation in KIRC was associated with favorable outcomes, challenging earlier reports suggesting its oncogenic role^30^,^32^. These findings may be influenced by differences in the tumor microenvironment or epigenetic regulation, such as promoter methylation^35^. Notably, FUT1 was highly expressed in BLCA and UCEC, marking the first report of its association with poor prognosis in these cancers. In LUAD, FUT1 expression was lower in tumor tissues compared to normal tissues, with high expression linked to better outcomes. This contrasts with studies reporting elevated serum FUT1 levels, which could be attributed to differences between mRNA and protein expression, as well as the potential secretion of FUT1 into the extracellular matrix or bloodstream, reflecting systemic changes rather than localized tumor expression^36^. In COAD and KIRC, FUT1 expression was stage-dependent, with higher levels in advanced-stage COAD and early-stage KIRC tumors^30^,^37^. Additionally, age-related differences in FUT1 expression were observed, with higher levels in younger KIRP and ESCA patients and older CHOL and THYM patients, suggesting potential age-specific roles.

Functional analysis indicated that FUT1 regulates cell adhesion, DNA repair, inflammation, and immune signaling. Experimental validation confirmed the role of FUT1 in promoting tumor cell invasion, migration, and proliferation. These findings align with reports that glycosylation mediated by FUT1 enhances β1 integrin-dependent cell adhesion in BLCA^38^,^39^. FUT1 also correlated with immune-related markers, including TMB and MSI, in several cancers. These associations suggest that FUT1 might influence tumor immunogenicity and predict responses to immune checkpoint blockade (ICB) therapy. The strong correlation between FUT1 expression and ICB gene expression in BRCA and THCA aligns with the clinical efficacy of ICB therapy in these cancers^40^,^41^. Furthermore, FUT1 was linked to TME components, particularly stromal and immune scores, across multiple cancers, supporting its role in immune cell infiltration and tumor progression.

FUT1 and its interacting proteins are primarily involved in critical metabolic pathways, as indicated by GO and KEGG enrichment analyses. These pathways are essential in tumor progression and in determining the response to immune therapy^42^. Glycosylation, the addition of sugar chains to proteins or lipids, can be categorized into N-glycosylation and O-glycosylation. N-glycosylation attaches sugars to asparagine or glutamine residues, while O-glycosylation attaches sugars to serine or threonine residues^43^. Sialylation, a specific form of glycosylation, occurs as a modification at the terminal ends of existing sugar chains. While all sialylation is a form of glycosylation, not all glycosylation involves sialylation^44^. Interestingly, the PPI results suggest that FUT1 interacts with ST3GAL1 and ST3GAL4, enzymes involved in O-linked and N-linked sialylation, respectively. ST3GAL1 adds sialic acid to O-linked sugars, playing a crucial role in cell interactions and signaling, while ST3GAL4 modifies N-linked sugars, influencing glycoprotein functions and immune regulation^45^,^46^. This indicates that FUT1-mediated glycosylation is intricately linked with sialylation processes, suggesting a potential mechanistic connection between FUT1, sialylation, and cancer progression. This link may offer valuable insights into how FUT1 influences tumor biology, with potential implications for therapeutic strategies targeting glycosylation pathways in cancer.

To further explore the potential clinical applications of FUT1, we conducted an analysis of its association with drug sensitivity. Our findings suggest that higher FUT1 expression correlates with increased sensitivity to small molecule TKIs, including Ibrutinib, Afatinib, Osimertinib, Gefitinib, and Sapitinib. TKIs are a class of drugs that target EGFR, inhibiting its tyrosine kinase activity to prevent cancer cell proliferation. The glycosylation of EGFR, including deglycosylation, sialylation, and fucosylation, directly impacts its phosphorylation status and functionality^47^. For instance, the addition of sialic acid enhances the stability and activity of EGFR, thereby increasing its sensitivity to TKIs^48^. Given that FUT1 is involved in glycosylation processes, particularly in the sialylation and fucosylation of proteins, it is likely that FUT1 contributes to EGFR modification, thereby influencing TKI sensitivity. These findings suggest that FUT1 could serve as a potential biomarker to predict the efficacy of TKI therapies in cancer treatment. This opens up exciting possibilities for further research into FUT1 as a therapeutic target, particularly in the context of EGFR-targeted therapies. Exploring the mechanisms through which FUT1 influences drug sensitivity could lead to more personalized and effective treatment strategies, especially for patients with EGFR-mutant cancers. Furthermore, understanding how FUT1-mediated glycosylation impacts cancer therapy may reveal novel avenues for improving treatment outcomes and overcoming resistance to current therapies.

## Conclusions

This study provides the first comprehensive pan-cancer analysis of FUT1, revealing its variable expression across tumor and normal tissues. Our findings demonstrate significant associations between FUT1 expression, clinical prognosis, and DNA methylation, highlighting its potential as a stand-alone prognostic biomarker in multiple tumor types. FUT1 expression is also correlated with immune-related features, including immune cell infiltration, MSI, and TMB. These results suggest that FUT1 may influence tumor immunity differently across cancer subtypes, warranting further investigation into its precise roles in tumorigenesis.

While this study provides valuable insights into the biological and clinical relevance of FUT1, several limitations should be noted. First, reliance on publicly available datasets may result in biases due to data heterogeneity and variability. Second, *in vitro* experiments cannot fully capture the complexity of tumor-immune interactions observed *in vivo*. Lastly, the mechanisms underlying the gene's role in immune modulation, TMB, and MSI remain inadequately understood. Future studies should validate these findings in clinical cohorts and further investigate its potential as a therapeutic target and modulator of the tumor microenvironment.

In summary, our results shed light on the multifaceted role of FUT1 in cancer progression and immunity, providing a foundation for developing targeted therapies and improving cancer treatment strategies.

## Supplementary Material

Supplementary figures and tables.

## Abbreviations

FUT4: Fucosyltransferase 4
FUT1: Fucosyltransferase 1
TMB: Tumor mutational burden
MSI: Microsatellite instability
OS: Overall survival
DSS: Disease-specific survival
DFI: Disease-free interval
PFI: Progression-free interval
HR: Hazard ratios
CI: Confidence intervals
R: Responders
NR: Non-responders
AUC: Area under the curve
CNVs: Copy number variations
SCNAs: Somatic copy number alterations
EMT: Epithelial-mesenchymal transition
PPI: Protein-protein interaction
TME: Tumor microenvironment
ICIs: Immune checkpoint inhibitors
IC50: Half-maximal inhibitory concentration
TKIs: Tyrosine kinase inhibitors
ICB: Immune checkpoint blockade
