# Supplementary material for: Comprehensive Multi-Omics Analysis Identifies FUT1 as a Prognostic and Therapeutic Biomarker Across Pan-Cancer
Source: Int J Med Sci. 2025 Feb 18;22(6):1313–28. doi: 10.7150/ijms.108072 (PMC11898856; doi:10.7150/ijms.108072)
Supplement: Supplementary file 1 — Supplementary figures and tables. [file ijmsv22p1313s1.pdf]

Supplementary materials

Supplementary Figures

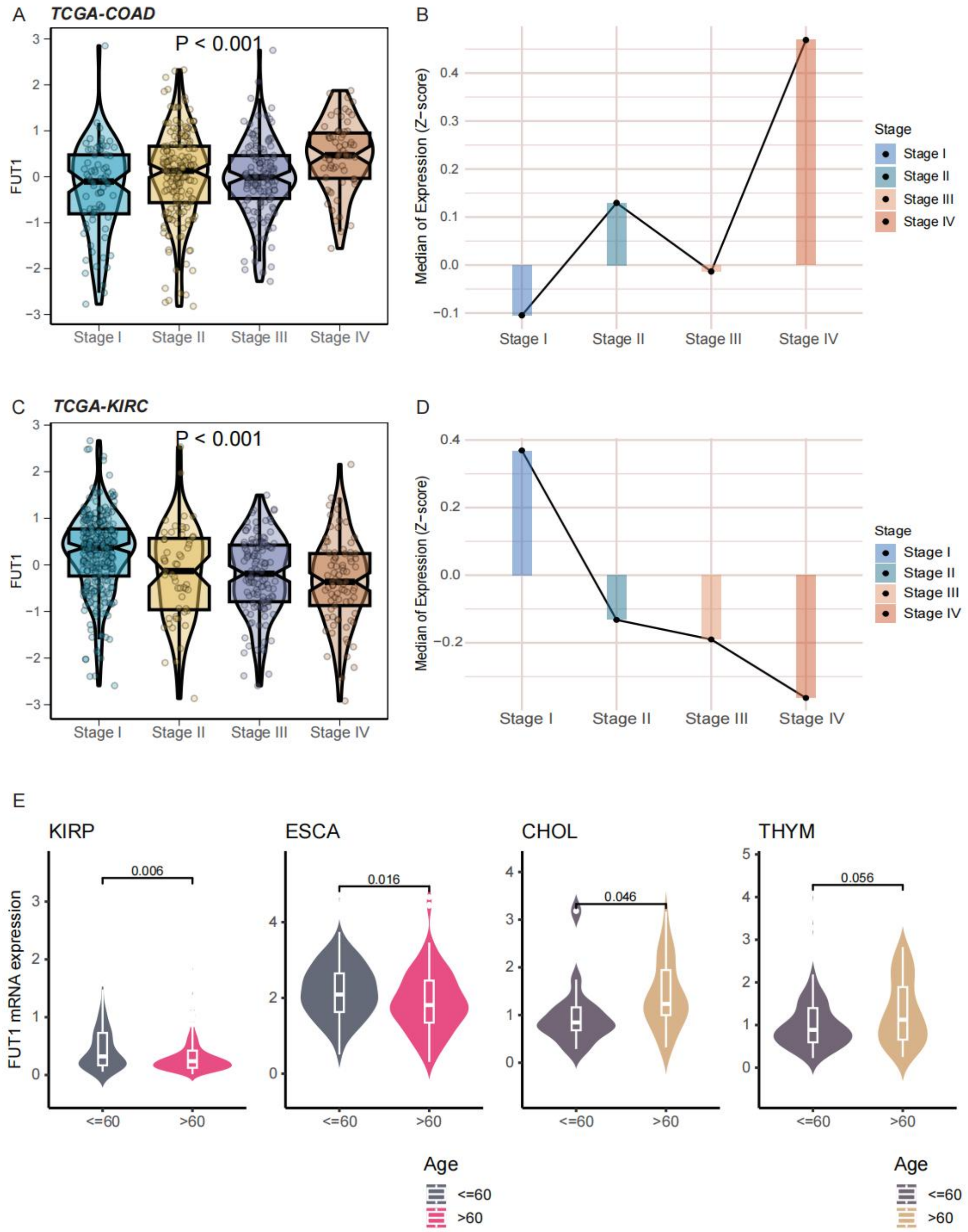

Figure S1. Differential expression of FUT1 mRNA across tumor stages and age groups in various cancer types. (A-D) FUT1 mRNA expression levels across different clinical stages (Stage I-IV) in various cancer types. Data are presented as Z-scores of mRNA expression, showing significant variation in FUT1 expression between stages (e.g., TCGA-COAD, TCGA-KIRC). (E-H) FUT1 mRNA expression levels stratified by patient age (≤60 vs. >60 years) across selected cancers (e.g., KIRP, ESCA, CHOL, THYM).

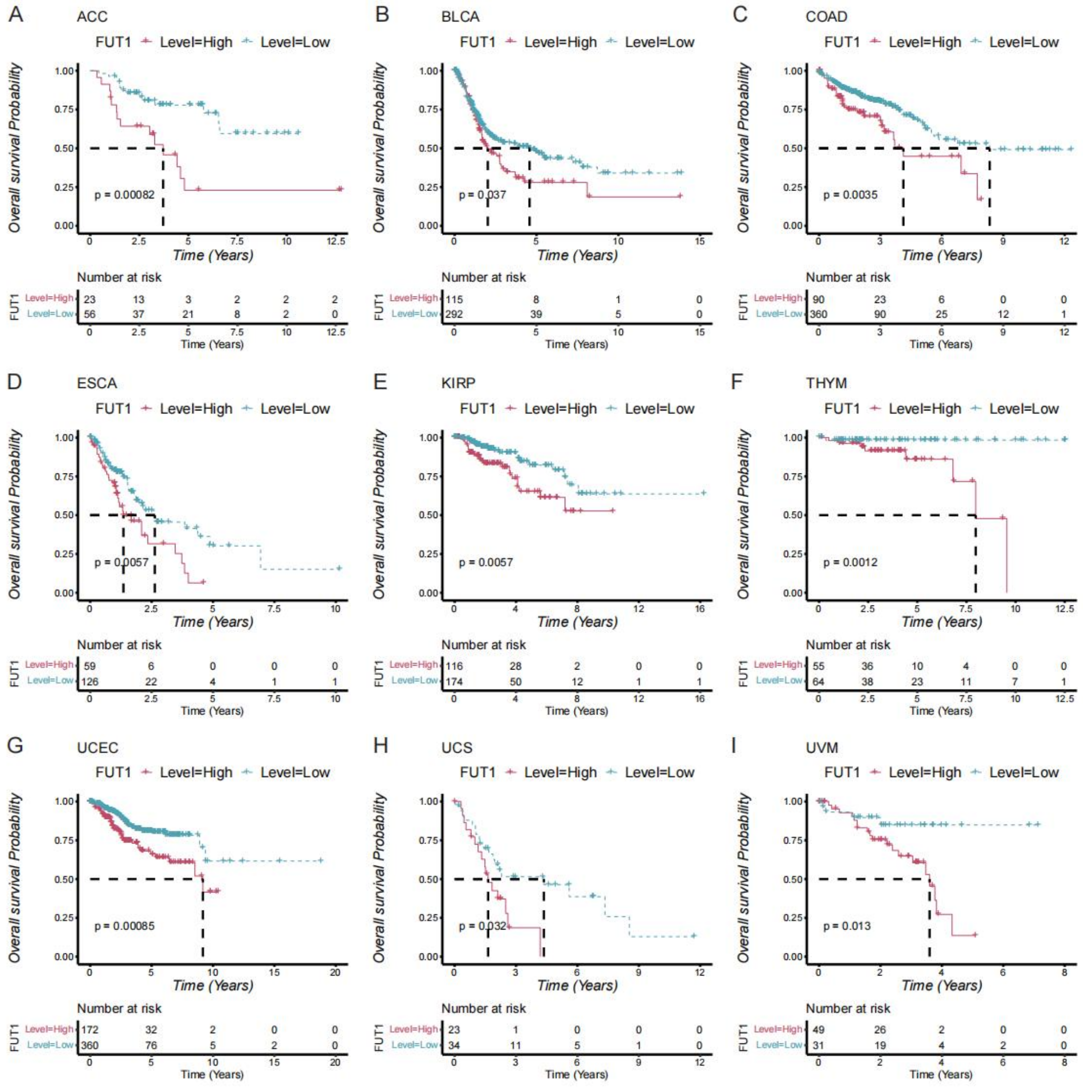

Figure S2. Kaplan-Meier analysis of Overall Survival (OS) based on FUT1 expression levels. Kaplan-Meier survival curves showing the overall survival (OS) differences between high and low FUT1 expression groups across various cancers. Survival data were derived from TCGA, and statistical significance was determined using the log-rank test.

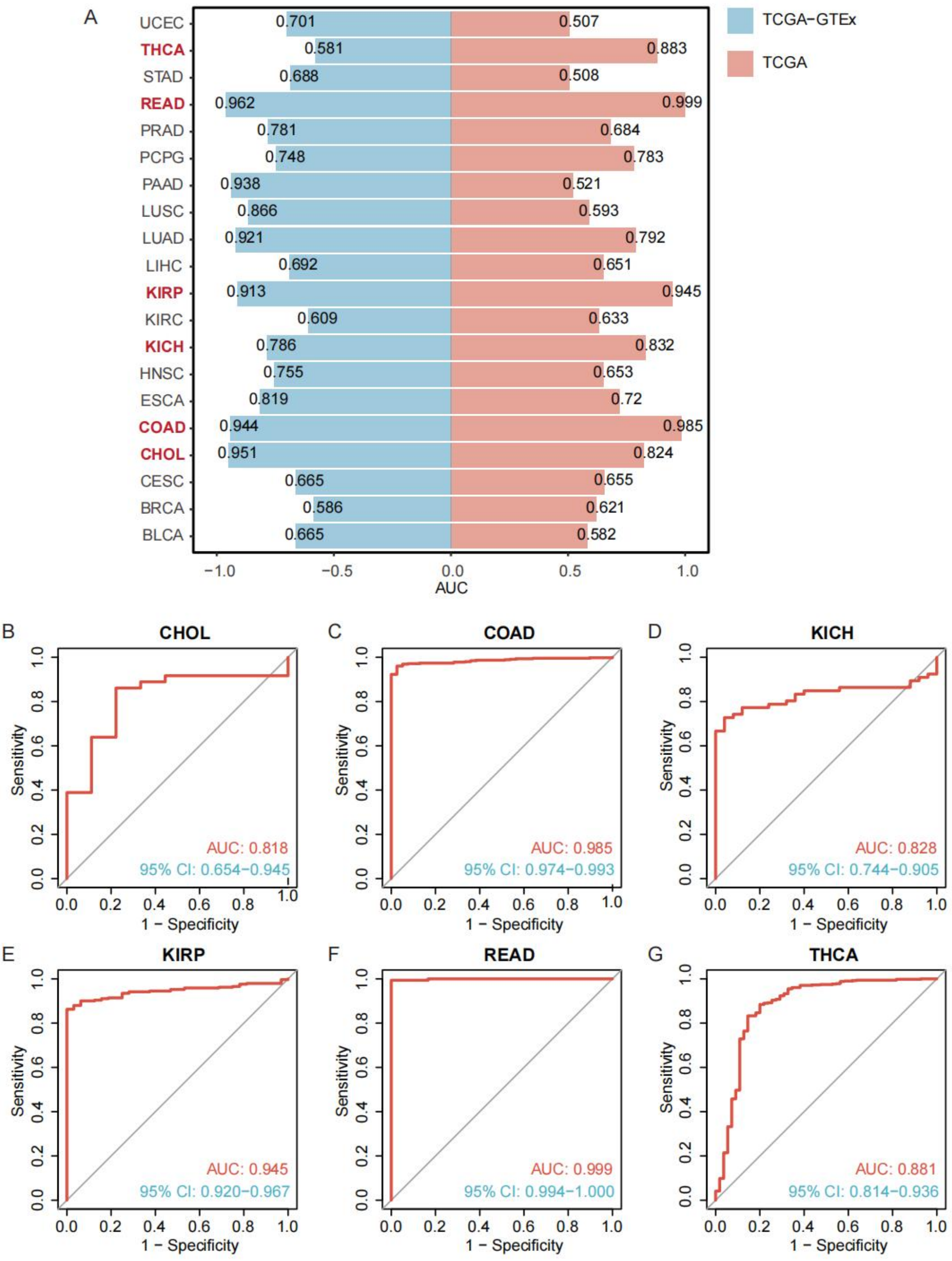

Figure S3. ROC analysis evaluating the diagnostic performance of FUT1 expression in tumor and normal Groups. Receiver operating characteristic (ROC) curves assessing the diagnostic ability of FUT1 expression to distinguish tumor tissues from normal tissues across multiple cancer types. Area under the curve (AUC) values with corresponding 95% confidence intervals (CI) are shown for each cancer type, based on data from TCGA and TCGA-GTEx.

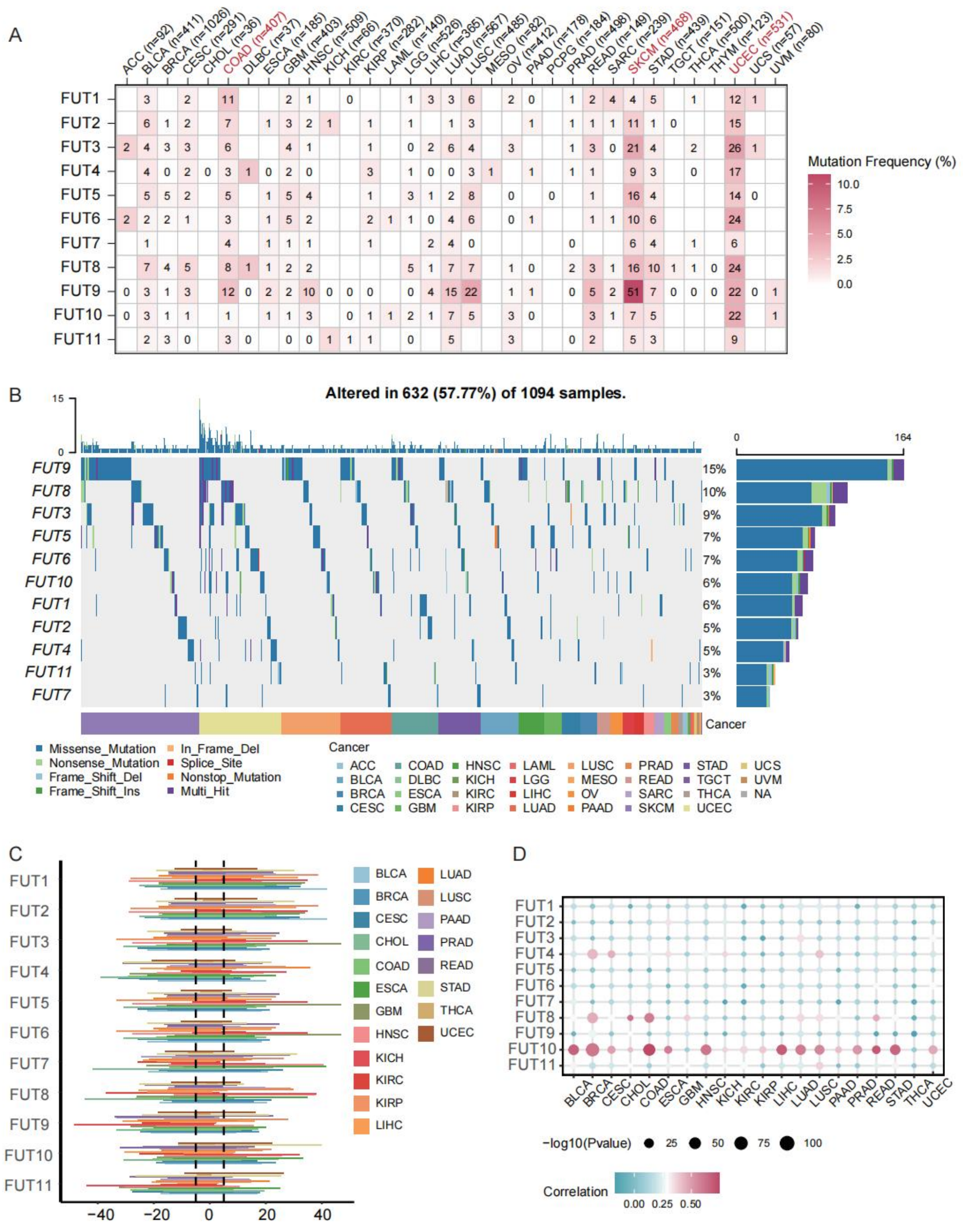

Figure S4. Genomic alterations of FUT Genes across pan-cancer
(A) Profile of single nucleotide variations (SNVs) in FUT genes across various cancer types, including mutation frequencies and types. (B) Waterfall plot showing the distribution of FUT1 mutations across pan-cancer. (C) Histogram displaying the frequency of somatic copy number alterations (SCNAs) for each FUT gene across different cancer types. (D) Spearman correlation analysis between somatic copy number alterations and FUT gene expression levels across cancers.

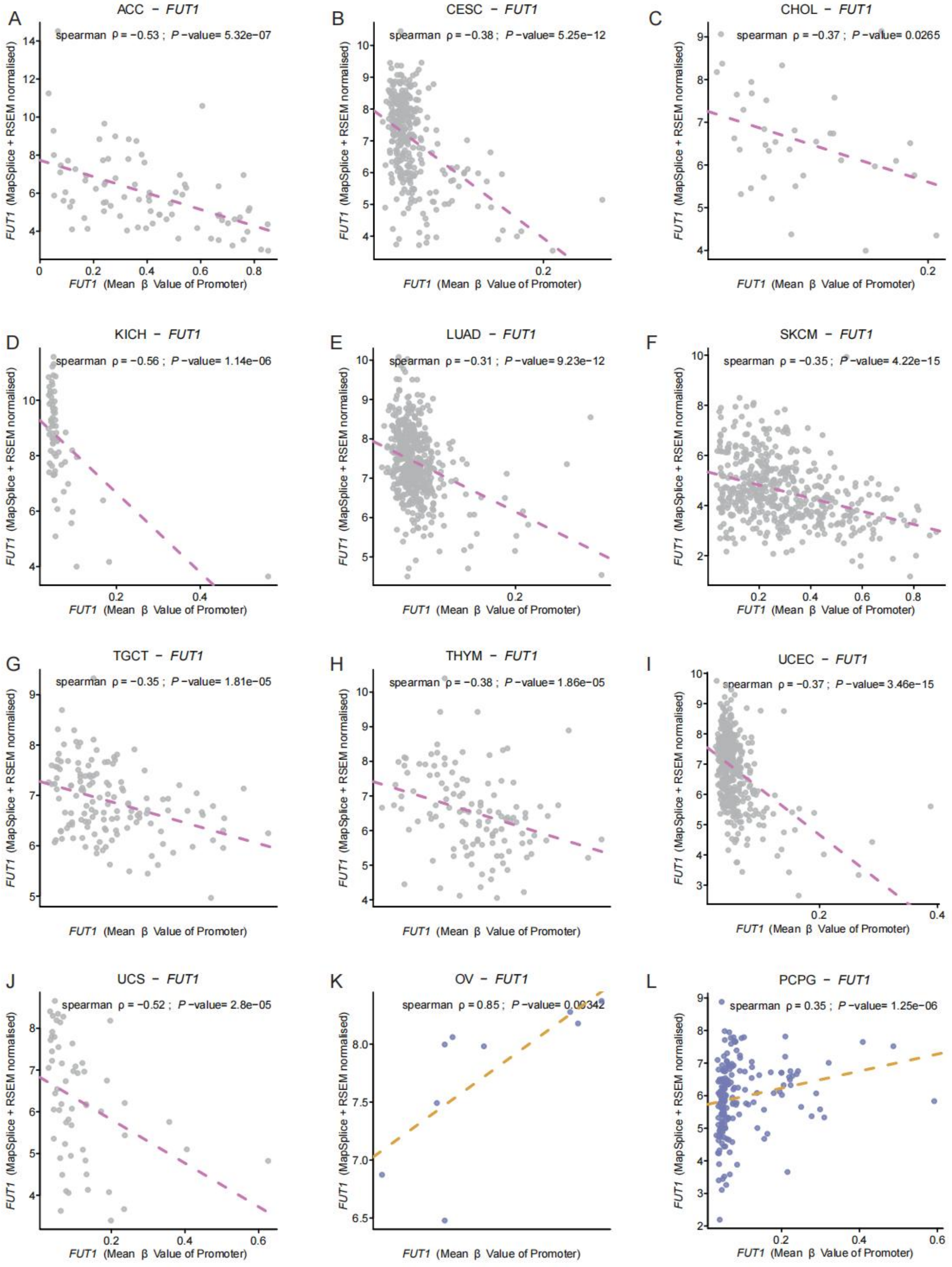

Figure S5. Correlation between FUT1 expression and DNA methylation levels across cancers. Scatter plots showing the correlation between FUT1 mRNA expression levels and DNA methylation levels (mean β value of promoter) in various cancer types. Spearman correlation coefficients (ρ) and p-values are indicated for each plot.

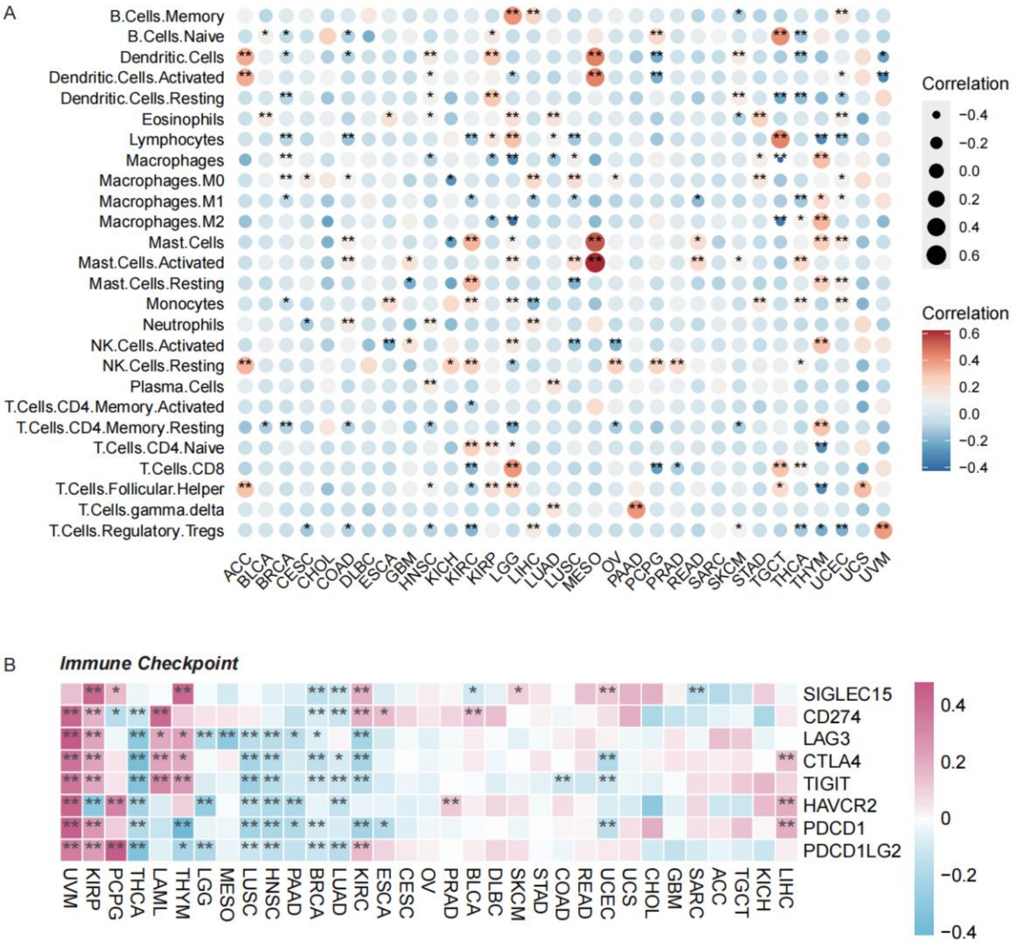

Figure S6. Association between FUT1 expression and immune cell infiltration and immune checkpoints. (A) The relationship between FUT1 expression and the infiltration levels of 26 immune cell types across cancers. Spearman coefficients and statistical significance values are provided to illustrate the association. (B) The connection between FUT1 expression and the expression levels of immune checkpoint genes, including PDCD1 (PD-1), CD274 (PD-L1), CTLA4, and others. Spearman analysis results are presented to highlight these relationships.

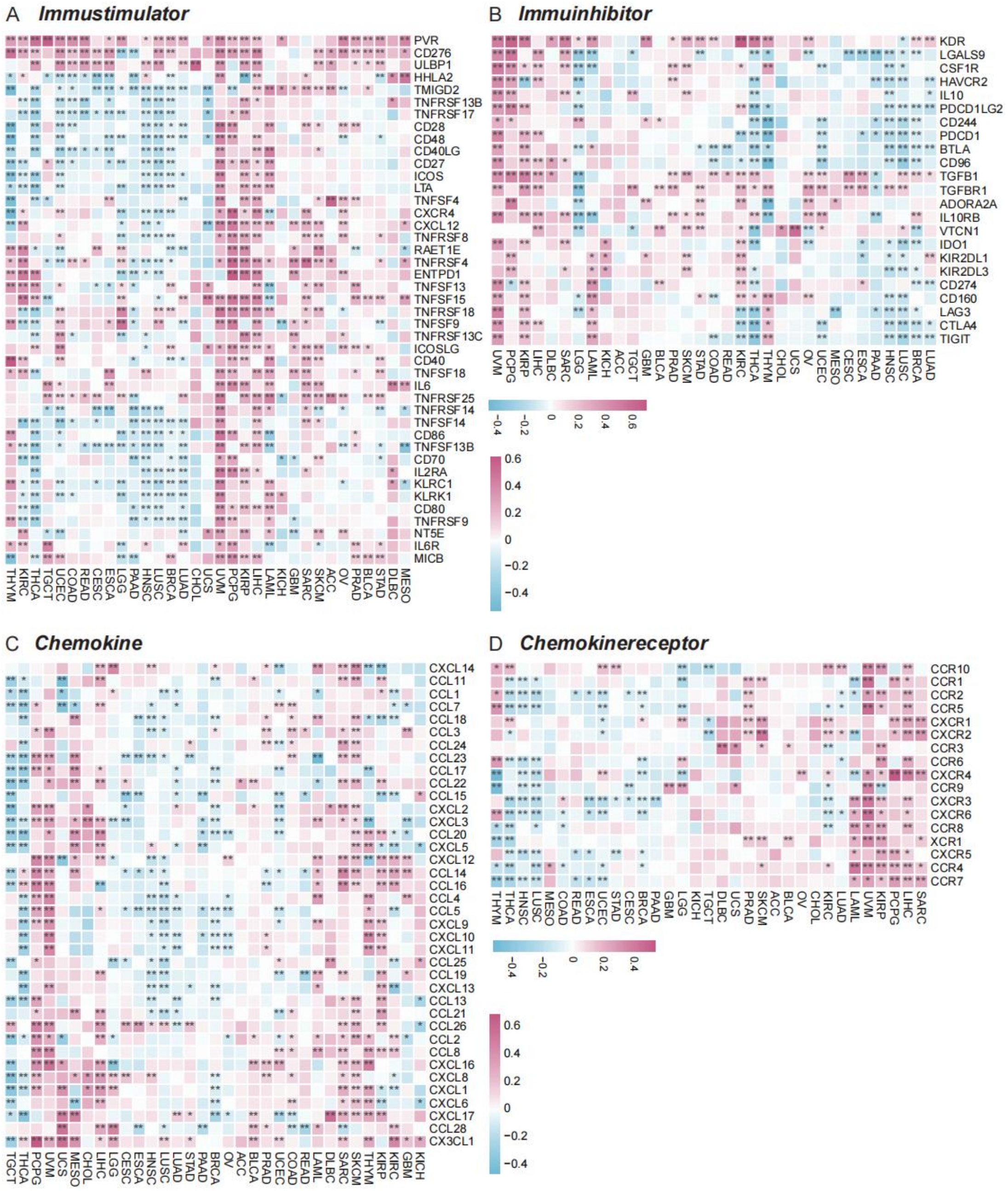

Figure S7. Correlation between FUT1 expression and immune-related genes in cancer. (A-D) Heatmaps illustrating the relationship between FUT1 expression and immune-related gene sets across multiple cancer types, including immune inhibitors (A), immune stimulators (B), chemokines (C), and chemokine receptors (D).

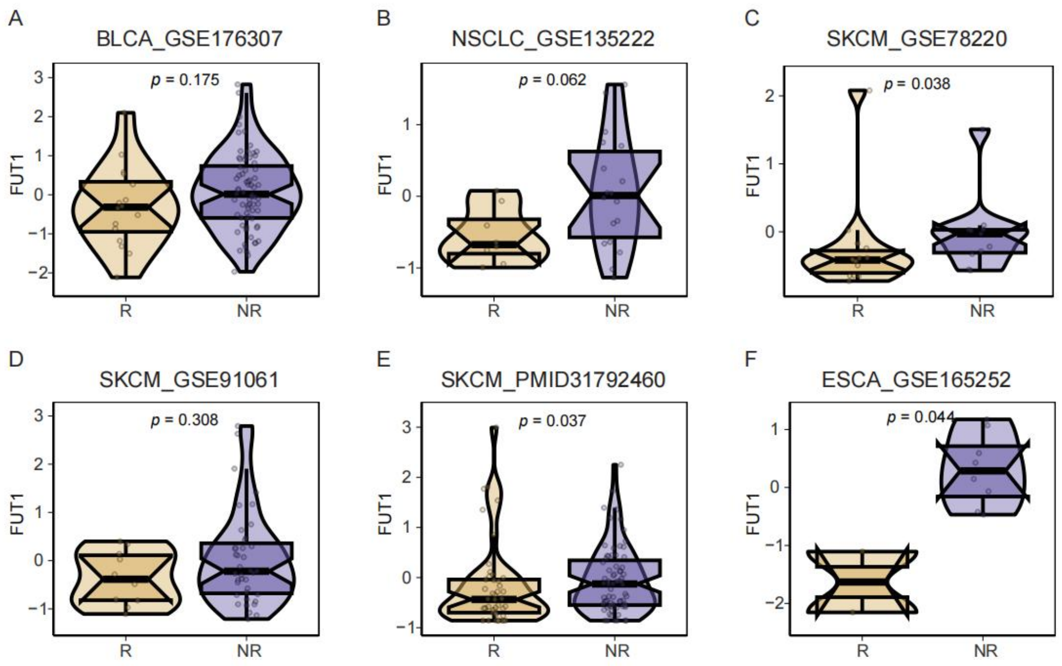

Figure S8. Differential expression of FUT1 between responders and non-responders in immune therapy cohorts. (A-F) Show the differential expression between R and NR groups in immune therapy cohorts for BLCA, NSCLC, SKCM, and ESCA.

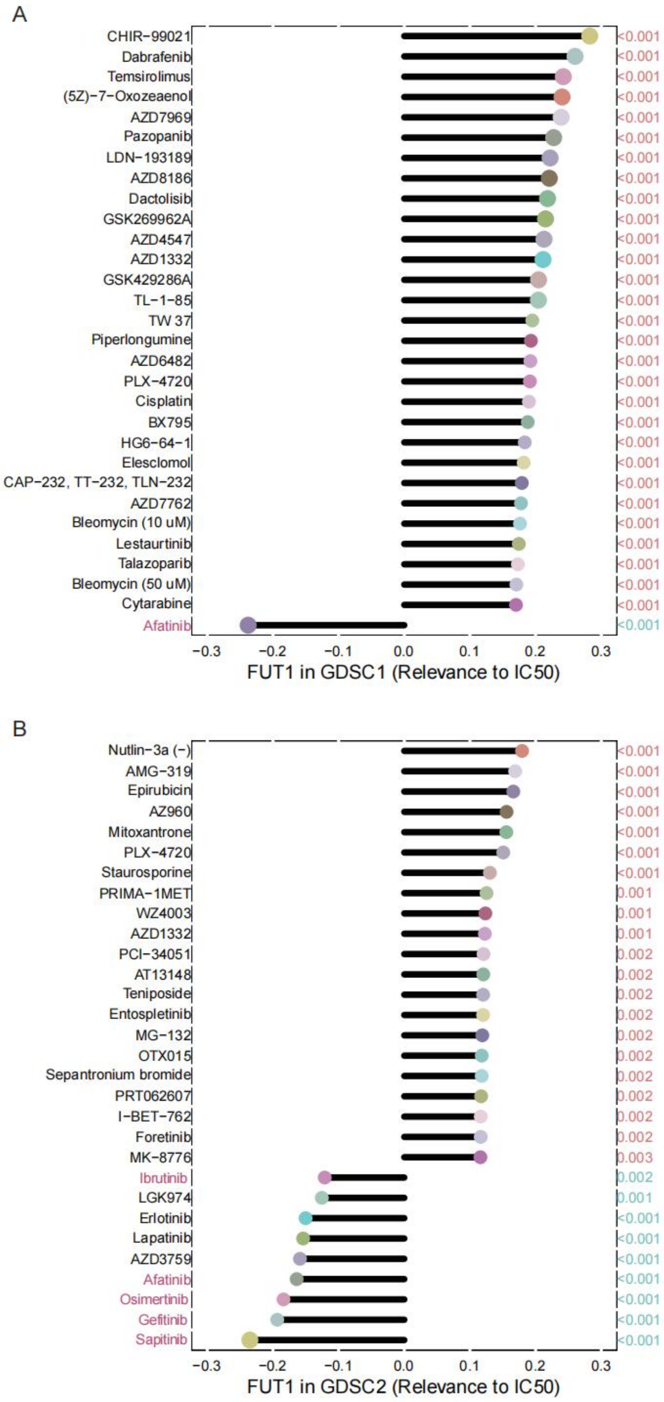

Figure S9. FUT1 as a predictive biomarker for treatment response. (A) Drug prediction based on GDSC1, showing the relevance of FUT1 expression to the IC50 values of various compounds. (B) Drug prediction based on GDSC2, illustrating the relationship between FUT1 expression and the IC50 values of additional compounds.

Supplementary Tables

Table S1. List of abbreviations.

**Abbreviations**	**Full name**
ACC	Adrenocortical carcinoma
ALL	Acute Lymphoblastic Leukemia
BLCA	Bladder Urothelial Carcinoma
BRCA	Breast invasive carcinoma
CESC	Cervical squamous cell carcinoma and endocervical adenocarcinoma
CHOL	Cholangiocarcinoma
CLL	Chronic Lymphocytic Leukemia
COAD	Colon adenocarcinoma
COADREAD	Colon adenocarcinoma/Rectum adenocarcinoma Esophageal carcinoma
DLBC	Lymphoid Neoplasm Diffuse Large B-cell Lymphoma
ESCA	Esophageal carcinoma
GBM	Glioblastoma multiforme
HNSC	Head and Neck squamous cell carcinoma
KICH	Kidney Chromophobe
KIRC	Kidney renal clear cell carcinoma
KIRP	Kidney renal papillary cell carcinoma
LAML	Acute Myeloid Leukemia
LCML	Chronic Myelogenous Leukemia
LGG	Brain Lower Grade Glioma
LIHC	Liver hepatocellular carcinoma
LUAD	Lung adenocarcinoma
LUSC	Lung squamous cell carcinoma
MESO	Mesothelioma
MM	Multiple Myeloma
NB	Neuroblastoma
OV	Ovarian serous cystadenocarcinoma
PAAD	Pancreatic adenocarcinoma
PCPG	Pheochromocytoma and Paraganglioma
PRAD	Prostate adenocarcinoma
READ	Rectum adenocarcinoma
SARC	Sarcoma
SKCM	Skin Cutaneous Melanoma
STAD	Stomach adenocarcinoma
STES	Stomach and Esophageal carcinoma
TGCT	Testicular Germ Cell Tumors
THCA	Thyroid carcinoma
THYM	Thymoma
UCEC	Uterine Corpus Endometrial Carcinoma
UCS	Uterine Carcinosarcoma
UVM	Uveal Melanoma

Table S2. Immunotharepy Cohorts and Data Source Information.

Cancer Type	Sample Type	Source	Treatment	Treatment Phase
SKCM	TILs	GSE100797	ACT	Pre
SKCM	Tumor	GSE145996	anti-PD1	Pre
SKCM	Tumor	GSE35640	MAGE-A3	Pre
SKCM	Blood	GSE106128	DCs	Pre
SKCM	Blood	GSE106128	DCs	Post
SKCM	Blood	GSE106128	DCs	1st
SKCM	Blood	GSE106128	DCs	2nd
SKCM	Blood	GSE106128	DCs	3rd
SKCM	Blood	GSE106128	DCs	4th
SKCM	Tumor	GSE78220	anti-PD1	Pre
SKCM	Tumor	GSE91061	anti-PD1	Pre
SKCM	Tumor	GSE91061	anti-PD1	On
SKCM	Tumor	PMID31792460	anti-PD1	Pre
SKCM	Tumor	PMID27956380	anti-CTLA4	Post
SKCM	Tumor	PMID27956380	anti-CTLA4	Pre
SKCM	Tumor	PMID30753825	anti-PD1	EDT
SKCM	Tumor	PMID30753825	anti-PD1	Pre
SKCM	Tumor	PMID30753825	anti-PD1-CTLA4	EDT
SKCM	Tumor	PMID30753825	anti-PD1-CTLA4	Pre
BLCA	Tumor	IMvigor210	anti-PD1	Pre
BLCA	Tumor	GSE176307	anti-PD1	Pre
KIRC	Tumor	PMID32472114	anti-PD1	Pre
KIRC	Tumor	GSE67501	anti-PD1	Pre
ESCA	Tumor	GSE165252	anti-PD1	Pre
ESCA	Tumor	GSE165252	anti-PD1	On
ESCA	Tumor	GSE165252	anti-PD1	Post
STAD	Tumor	PMID30013197	anti-PD1	Pre
BRCA	Tumor	GSE139050	anti-PD1	Pre
NSCLC	Tumor	GSE126044	anti-PD1	Pre
NSCLC	Tumor	GSE135222	anti-PD1	Pre
MESO	Tumor	GSE99070	anti-PD1	Pre
